# 

**Published:** 1886-07-20

**Authors:** 


### EDITORIAL NOTICES.

**Something Useful.**—To any one who will send us four cash subscribers, we will send as a premium one of Barry's splendid Chemical Thermometers.

R. C. WORD, *Managing Editor.*

☞ **For Sale.**—A scholarship of Business College at Louisville, Ky., at reduced rates. Inquire of R. C. Word, Managing Editor of this journal.

THE SOUTHERN MEDICAL COLLEGE will commence its next Course of Lectures on the 6th of October next. The introductory address will be delivered by Dr. G. G. Roy, Professor of Materia Medica in the Institution.

GYNÆCOLOGY IN THE SOUTHERN MEDICAL COLLEGE.—Virgil O. Hardon, M. D., has been appointed Lecturer on Operative Gynæcology in the Southern Medical College, for which position he is eminently qualified, being a gentleman of fine acquirements, and having been a long while associated with Dr. J. Marion Sims, M. D., LL. D., the distinguished Gynæcologist of New York.

DR. G. C. SAVAGE, of Jackson, Tenn., has been elected to the chair of Diseases of Eye and Ear in the Medical Department of Vanderbilt University.

A NEW medical society, entitled the Nashville Academy of Medicine, has been inaugurated in that city. An inaugural was delivered on the occasion by Dr. J. W. Maddin.

THE NATIONAL ECLECTIC MEDICAL ASSOCIATION held its last annual meeting in Atlanta, from the 16th to the 18th of June. It was spoken of as one of the largest meetings ever held by that body.

DR. J. L. HAMILTON, vice-president of the Stone Mountain Medical Association, is a candidate for Senatorial honors. The Doctor is a good man, and, if elected, will do no harm to the country. We think a good doctor ought to make a good legislator.

WE learn that Dr. Hunter P. Cooper, formerly of New York City, has been appointed Adjunct Professor of Chemistry in the Atlanta Medical College. Dr. Cooper stands high in the profession, and his appointment is regarded as a fortunate one for the College.

AN OLD PROFESSOR.—Dr. Joseph Neyron is believed to be the oldest acting medical professor in the World, being in the chair of Anatomy and Medicine in the University of Notre Dame, Indiana. He is also a Catholic priest, and was a surgeon at the battle of Waterloo, in the division of Marshal Ney that deserted to Napoleon. Although ninety six years old, he is possessed of a good degree of physical vigor, and of undiminished mental powers.

MONTEAGLE. - Our Senior Editor, Dr. Thomas S. Powell, made an address, on the 8th instant, to a large and appreciative audience, at the above celebrated resort, which is also called the Chautaqua of the South. The address was highly commended, as one of the ablest and best ever delivered at Monteagle. Doctor Powell had a very agreeable sojourn at Monteagle, and returns to his home refreshed and invigorated for the duties before him.

PROF. J. MCF. GASTON, of the Southern Medical College, Atlanta, has been invited to contribute a paper to the next British Medical Association. The Surgical Relations of the Gall-bladder, the subject upon which he is to write, is one to which he has devoted considerable study, having proven by operations upon the lower animals the possibility, in cases of obstruction in the common duct, of establishing a fistulous communication between the gall-bladder and intestine.

MALTINE WITH CASCARA.—This is a new combination, prepared by the Maltine Manufacturing Company. It would seem to be especially adapted to that form of indigestion, so common, in which there is constipation with torpid condition of the lower and upper bowels—a condition which is frequently associated with portal congestion, and hemorrhoidal tumors, etc.—as it is laxative, tonic and alterative. The Maltine Manufacturing Co , 182 Fulton street, New York, will send a sample for trial to any physician who will pay the express charges on the same.

THE EVENING CAPITAL.—This is a paper published in Atlanta. The business management is in charge of Charles S. Atwood, a gentleman of great business tact and energy. The editorial manager is Col. I. W. Avery, a gentleman well known in Georgia as formerly connected with the Executive Department during Governor Colquitt's administration, as the author of an important historical work, and as a writer of eminent ability. The paper is issued every evening ; has now a very large circulation, and is in all respects a newsy and interesting sheet.

PROGRESS is the name of a new medical journal published in Louisville, Ky., and edited by Dudley S. Reynolds, A. M., M. D. The number before us is ably gotten up, and presents a neat appearance. Success to brother Reynolds. He mentions editorially that he has extracted ninety-three cataracts under the use of a solution of cocaine, prepared by dissolving 2 grains of the muriate of cocaine in a drachm of distilled water. The results were highly satisfactory—only one failure, and that in an old man, who injured the eye after the operation. The noxious properties attributed to cocaine by Dr. Calhoun he has not found in his experience.

SALT SPRINGS.—Salt Springs, located in the immediate vicinity of Austell, Ga., west of Atlanta, near the depot of the East Tennessee, Virginia and Georgia Railroad and Georgia Pacific Railway, is now attracting much attention for the remarkable efficacy of the waters. Our Managing Editor will, if possible, before the next issue of the RECORD do himself the pleasure of visiting this noted health resort, to examine the place and the water, and test its medicinal powers, being greatly in need of rest and recreation by reason of sedentary habits and protracted office labors. On his return, he will inform our readers in relation to the locality and the medicinal qualities of the water.

BENZOL SULPHURICIMIDE.—This is a new sacharine substance produced from coal tar. The name *sacharine* has been most frequently applied to it. It was discovered by Dr. Fahlberg. It is a white powder, and is intensely sweet, far surpassing the best cane sugar, or any substance known. It is slightly antiseptic, and will probably be applied to many useful purposes. Its present price is 50 cents per pound, but as it is ten times sweeter than cane sugar, it is comparatively cheap as a sweetening agent. It is said to be wholesome, but its properties as a medicine, etc., are not yet fully known.

